# A scoping review of models for predicting the risk of postherpetic neuralgia

**DOI:** 10.3389/fmed.2025.1653680

**Published:** 2025-10-03

**Authors:** Lifeng Zhang, Nan Qu, Tiantian Li, Lizhen Duan, Liping Cui

**Affiliations:** ^1^Department of Nursing, Shanxi Bethune Hospital, Shanxi Academy of Medical Sciences, Third Hospital of Shanxi Medical University, Tongji Shanxi Hospital, Taiyuan, China; ^2^School of Nursing, Shanxi University of Chinese Medicine, Jinzhong, China

**Keywords:** postherpetic neuralgia, risk assessment, prediction model, scoping review, herpes zoster (HZ)

## Abstract

**Objective:**

To conduct a scoping review of risk prediction models for postherpetic neuralgia (PHN), providing insights for clinical identification of patients at high risk and future research.

**Methods:**

China National Knowledge Infrastructure, Wanfang, VIP Database, Chinese Biomedical Literature Service System (SinoMed), PubMed, Embase, Web of Science and the Cochrane Library databases were systematically searched from database establishment to 25 October 2024, and data on the prevalence of PHN, model construction, predictors and model performance were extracted for summary analysis.

**Results:**

A total of 23 studies were included, with a high overall risk of bias. The prevalence of PHN ranged from 6.20 to 48.00%, with traditional logistic regression being the predominant model construction method. The three most frequently identified predictive factors were age, rash area and pain severity score. Additionally, 43.48% of the studies did not validate their models, and 52.17% used visualization methods to present their models. The area under the receiver operator characteristic curve of the studies was 0.714–0.980. Two studies performed external validation; 14 studies evaluated the model’s calibration, and the calibration curve coincided well with the actual curve; and eight studies assessed the clinical benefit.

**Conclusion:**

Risk prediction models for PHN all showed good predictive performance, but the risk of bias was high, and further clinical validation is needed. In the future, research could refine variable selection and model performance evaluation to optimize predictive models continuously, aiming to develop models with excellent predictive performance and strong clinical utility.

**Systematic review registration:**

DOl: https://doi.org/10.17605/0SF.IO/SUR2C.

## 1 Introduction

Postherpetic neuralgia (PHN), the most prevalent complication of herpes zoster (HZ), manifests as a complex neuropathic pain syndrome (1), characterized by spontaneous or episodic pain that may endure for months, years or even a lifetime. Postherpetic neuralgia not only exerts a profound impact on patients’ sleep quality, physical sensation and psychological well-being but also imposes considerable economic burdens (2). In China, the prevalence of HZ stands at 7.7%, with 29.8% of affected individuals progressing to PHN (3). Both the prevalence and severity of PHN increase with advancing age. Nevertheless, treatments for PHN frequently yield less than satisfactory outcomes, as fewer than half of patients experience a 50% or greater reduction in pain intensity (4). Consequently, early identification and timely intervention for patients at high risk of PHN are of paramount importance. With the advent of the digitally intelligent healthcare era, clinical predictive models have seen substantial expansion in application across medical diagnostics, treatment plan selection and patient prognosis management (5). Several researchers have developed predictive models to identify patients at high risk of PHN. Nevertheless, whether discrepancies exist in model construction methodologies, performance and predictive factors remains to be investigated. Consequently, in accordance with the scoping review framework proposed by Arksey and O’Malley (6), this study undertakes a systematic analysis and synthesis of existing PHN risk prediction models, aiming to facilitate the implementation of PHN secondary prevention strategies in clinical practice and to guide future research.

## 2 Materials and methods

### 2.1 Research questions

(1) What PHN risk prediction models are currently available? (2) What methodologies are utilized for model construction? (3) Which predictive factors are incorporated into these models? (4) What is the predictive performance of these models? This study has been registered on the Open Science Framework (doi: 10.17605/OSF.IO/SUR2C).

### 2.2 Literature search

A comprehensive search was conducted across multiple databases, including China National Knowledge Infrastructure, Wanfang, VIP Database, Chinese Biomedical Literature Service System (SinoMed), PubMed, Embase, Web of Science and the Cochrane Library, from inception to 25 October 2024. The search terms used were in both Chinese and English, covering herpes zoster, herpes zoster virus infection, herpetic neuralgia, PHN, postherpetic pain, postherpetic sequelae, postherpetic neuropathy, postherpetic chronic pain, risk assessment, risk prediction, risk factors, prediction model, prediction, model and nomogram. The search was executed via a hybrid approach combining subject terms and free-text terms. For specific strategies, see Supplementary material.

### 2.3 Inclusion and exclusion criteria

The inclusion criteria were as follows: (1) study population: patients diagnosed with HZ; (2) study content: construction or validation of PHN risk prediction models; (3) study design: prospective or retrospective studies (including cross-sectional, case–control and cohort studies); (4) articles published in peer-reviewed journals or academic dissertations in either Chinese or English. The exclusion criteria were as follows: (1) duplicate publications (including those overlapping with master’s or doctoral theses) and (2) studies with inaccessible full texts.

### 2.4 Literature selection and data extraction

Duplicate literature entries were first removed using the NoteExpress software (Beijing E-Cheng Qinghua Technology Development Co., Ltd., Beijing, China). Two independent investigators conducted an initial screening of titles and abstracts based on the pre-established inclusion and exclusion criteria. Subsequently, a full-text review was conducted to finalize the literature included. Any discrepancies that arose during the screening process were resolved by seeking input from a third investigator. Data extraction was performed using a standardized data extraction form developed based on the Critical Appraisal and Data Extraction for Systematic Reviews of Prediction Modelling Studies (7) checklist, extracting information on variables such as publication year, country of the study, study population, data collection methods, sample size and PHN incidence rate.

### 2.5 Bias risk and applicability assessment

Two investigators independently assessed the risk of bias and the applicability of the included literature using the Prediction Model Risk of Bias Assessment Tool (8). This tool evaluates four main domains: participants, predictors, outcomes and analysis. Each domain is judged as low, high or uncertain. The evaluation criteria for each domain and our assessment methodology are as follows: participants: assess whether the study population is representative of the target population and whether selection bias is present; predictors: evaluate whether the measurements of predictors are accurate and consistent; outcomes: assess whether the definitions and measurements of outcomes are clear and consistent; analysis: evaluate whether statistical analysis methods are appropriate and whether there are issues such as overfitting.

For each domain, if all criteria are met, it is judged as low risk; if there is a serious problem, it is judged as high risk; if the information is insufficient, it is judged as uncertain risk. Any discrepancies were resolved by obtaining consensus through consultation with a third investigator.

### 2.6 Statistical analysis

The characteristics and outcomes of the included studies were analyzed using narrative summarization and descriptive methods.

## 3 Results

### 3.1 Literature selection process and results

**Figure 1:**
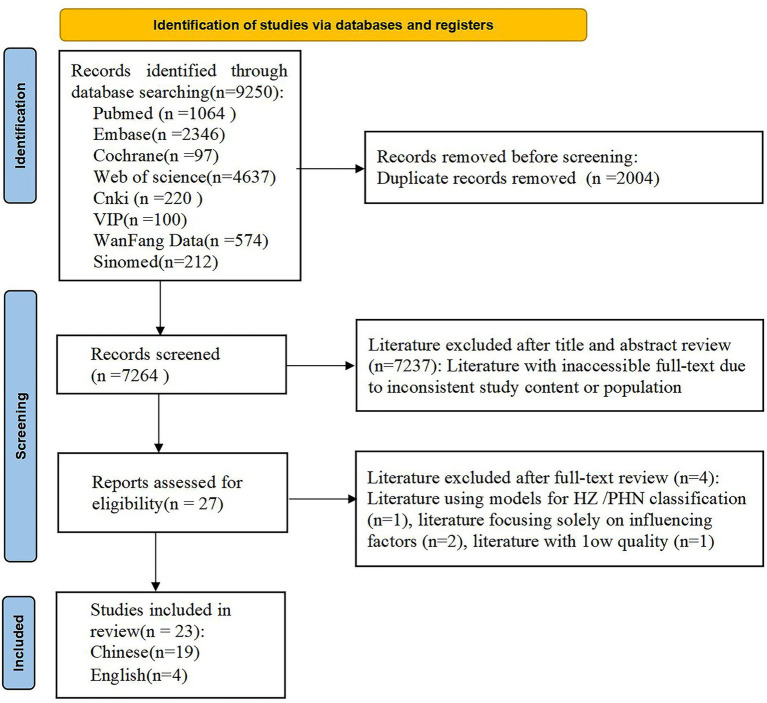
Literature selection process.

A total of 9,250 relevant pieces of literature were retrieved through the search, and the literature screening process is illustrated in Figure 1.

### 3.2 Basic characteristics of the literature included

A total of 23 studies were ultimately included. Of these, 17 studies were published between 2021 and 2024, four between 2010 and 2020 and two prior to 2010. Geographical distribution showed that 19 studies were from China, whereas 1 study each originated from Germany, the Netherlands, South Korea and Japan. Among the included studies, 60.86% used retrospective data, and 86.96% were single-center studies. The basic characteristics of the included literature are presented in Table 1 (9–31).

**Table 1:** Basic characteristics of the literature included.

Literature included	Publication year	Country	Study population	Data collection	Sample size	Study type	PHN incidence	Modeling method	Discriminant validity	Calibration	Clinical benefit	Validation method	Male/female	Age range (years)	Rash area (%)	Pain severity score
Meister et al. (9)	1998	Germany	HZ	Prospective	635	Cohort study	20.60%	①	—	—	—	Random split	279/356	/	/	/
Opstelten et al. (10)	2007	Netherlands	HZ (>50 years)	Prospective	598	Cohort study	7.70%	①	0.770^a^	0.76^c^	—	Bootstrap method	234/365	>50	0–47 skin lesions	0–100
Cho et al. (11)	2014	South Korea	HZ	Prospective	305	Cohort study	6.20%	①	0.868^a^	—	—	—	194/111	18–83	0–50 skin lesions	0–10
Hashizume et al. (12)	2022	Japan	HZ	Retrospective	79,264	Cohort Study	0.95%	①	0.616^b^	0.133^c^	—	Random split	29,522/49742	≥40	/	/
Lu and Cheng (13)	2015	China	HZ	Retrospective	220	Cohort study	17.30%	①	0.953 ± 0.014^a^	—	—	—	118/102	/	/	1–4
Li et al. (14)	2020	China	HZ	Retrospective	1,303	Cohort study	43.82%	②	0.752^a^	—	—	Random split	/	/	/	0–100
Wang et al. (15)	2020	China	HZ	Retrospective	502	Case–control study	24.90%	③④	0.980^a^	—	—	External validation	237/265	>0	/	0–10
Li et al. (16)	2022	China	HZ	Retrospective	425	Cohort study	30.12%	①	0.812^a^/0.824^b^	⑩	—	—	190/235	>18	0–5%	0–10
Liu et al. (17)	2022	China	HZ	Prospective	174	Cohort study	29.90%	①	0.810^a^	⑩	DCA	—	71/103	≥18	/	0–100
Lu et al. (18)	2022	China	HZ	Prospective	150	Cohort study	37.33%	⑤	0.769^b^	⑩	DCA	—	86/64	≤80	0–5%	0–100
Li (19)	2022	China	HZ	Retrospective	200	Case–control study	25.00%	①	0.820^a^ /0.820^b^	⑩	DCA	—	83/117	≥18	/	0–10
Zhang et al. (20)	2022	China	HZ	Retrospective	732	Cohort study	19.40%	①⑥	0.884^a^	None	—	Cross-validation	315/417	/	0–6	0–10
Lu et al. (21)	2023	China	HZ	Prospective	90	Cohort study	46.70%	①	0.910^a^	None	—	Random split	36/54	≥40	/	0–10
Mao et al. (22)	2023	China	HZ	Prospective	258	Cohort study	32.20%	①	0.897^a^	⑩	—	—	131/127	≥14	0–5%	0–10
Tian et al. (23)	2023	China	HZ	Retrospective	416	Cohort study	23.56%	①	0.789^a^	⑩	—	Random split	209/207	/	/	0–10
Wang et al. (24)	2023	China	HZ	Retrospective	307	Case–control study	32.80%	①	0.829^a^ /0.769^b^	0.168^c^	DCA	Bootstrap method	157/150	/	0–5%	0–10
Li et al. (25)	2023	China	HZ	Retrospective	198	Case–control study	28.28%	①	0.902^a^	0.628^c^	—	—	98/100	≥18	/	0–10
Yang et al. (26)	2024	China	HZ	Prospective	434	Cohort study	45.00%	①⑥⑦	0.860^a^	0.162^c^	DCA	Cross-validation	230/204	≥18	0–3/4	0–100
Zhao (27)	2023	China	HZ	Retrospective	889	Cohort study	30.60%	③④⑦	0.8140^a^	⑩	DCA	Bootstrap method, external validation	457/432	≥18	0–4	0–10
Liao et al. (28)	2023	China	HZ (treated with pulsed radiofrequency)	Prospective	50	Cohort study	48.00%	①	0.8165^a^	—	—	—	23/27	33–87	0–4	0–10
Tang et al. (29)	2024	China	HZ (combined with diabetes)	Retrospective	136	Case–control study	47.79%	①	0.714^a^	—	—	—	63/75	18–85	/	0–10
Lin et al. (30)	2024	China	HZ	Retrospective	524	Cohort study	43.70%	③④⑥⑦⑧⑨	0.820^a^	⑩	DCA	Cross-validation	238/286	>18	/	0–10
Cai et al. (31)	2024	China	HZ	Retrospective	209	Cohort study	29.67%	①	0.776^a^	⑩	DCA	Bootstrap method	130/79	/	/	0–10

Decision curve analysis (DCA); ^a^ denotes the AUROC value; ^b^ refers to the C-index; ^c^ represents the *p*-value of the Hosmer–Lemeshow test; ① logistic regression model; ② TREENET algorithm; ③ random forest; ④ logistic regression algorithm; ⑤ Cox proportional hazards model; ⑥ support vector machines (SVM); ⑦ eXtreme Gradient Boosting (XGBoost); ⑧ k-nearest neighbor algorithm; ⑨ neural network; ⑩ calibration curve.

### 3.3 Literature risk assessment

The included literature predominantly exhibited a high risk of bias, with 43.48% assessed as having a low overall risk of applicability. The assessment results are presented in Table 2.

**Table 2:** Literature risk assessment.

Literature included	Study population	Predictor factors	Outcomes	Analysis	Overall	Applicability
Meister et al. (9)	Low	Low	Low	High	High	Low
Opstelten et al. (10)	Low	High	Low	Low	High	High
Cho et al. (11)	Low	High	Low	High	High	High
Hashizume et al. (12)	Low	High	Low	High	High	High
Lu and Cheng (13)	Low	Unclear	High	High	High	High
Li et al. (14)	Low	High	Unclear	High	High	High
Wang et al. (15)	Unclear	Unclear	Low	High	High	Unclear
Li et al. (16)	Low	Low	Low	High	High	Low
Liu et al. (17)	Low	High	Unclear	High	High	High
Lu et al. (18)	Low	Unclear	High	High	High	High
Li (19)	Low	Low	Low	High	High	Low
Zhang et al. (20)	Low	High	Low	Low	High	High
Lu et al. (21)	Low	High	Low	High	High	High
Mao et al. (22)	Low	Low	Low	High	High	Low
Tian et al. (23)	Low	Low	Low	High	High	Low
Wang et al. (24)	Low	High	Low	High	High	High
Li et al. (25)	Low	Low	Low	High	High	Low
Yang et al. (26)	Low	Unclear	Low	High	High	Unclear
Zhao (27)	Low	Low	Low	High	High	Low
Liao et al. (28)	Low	Low	Low	High	High	Low
Tang et al. (29)	Low	Low	Low	High	High	Low
Lin et al. (30)	Low	Low	Low	High	High	Low
Cai et al. (31)	Low	High	Unclear	High	High	High

### 3.4 Overview of model construction

The populations of the studies included outpatients, hospitalized patients and community-based patients with HZ. Sample size: the sample size of the included studies ranged from 50 to 79,264 patients, with 34.78% of studies including more than 500 patients. Modelling methods: the methods employed included traditional logistic regression, Cox proportional hazards regression and machine learning algorithms. Five studies specifically utilized different methods (15, 20, 26, 27, 30).

### 3.5 Predictive factors of the models and their presentation formats

The number of predictive factors analyzed ranged from 2 to 10, and these were categorized into five types: general information, disease-related factors, treatment-related factors, laboratory indicators and other factors. The most common predictive factors were age, rash area and pain intensity. A total of 52.17% of the studies employed visualization to present the models. A detailed summary of the predictive factors in the models and their presentation formats is provided in Table 3.

**Table 3:** Predictive factors of the models and their presentation formats.

Literature included	Predictive factors (OR/β, 95% CI)	Presentation format	PROBAST overall applicability risk
Meister et al. (9)	Age, HZ type, prodromal pain, rash area, gender, site	①	Low
Li et al. (16)	Age (2.318, 1.438–3.735), diabetes (2.392, 1.513–3.781), smoking (2.202, 1.392–3.483), rash area (1.969, 1.244–3.115), VAS score (1.894, 1.191–3.012), CD4+/CD8 + ratio (2.247, 1.396–3.617)	⑤	Low
Li (19)	Prodromal pain (2.826, 1.199–6.152), rash area (1.002, 1.002–1.004), VAS score (10.265, 1.003–1.042), age (3.152, 0.995–9.213), female (2.936, 1.136–6.362)	⑤	Low
Mao et al. (22)	Age, initial treatment time, lesion area, statin medication history (3.53, 1.520–8.198), underlying diseases (2.77, 1.125–6.821), NSE (1.616, 1.223–2.134), TG (1.501, 1.004–2.244), VAS score	⑤	Low
Tian et al. (23)	60 years and above (3.100, 1.144–9.892), prodromal pain (2.099, 1.227–3.663), early treatment time (2.684, 1.587–4.599), blood CRP level (1.676, 1.436–1.981)	None	Low
Li et al. (25)	No glucocorticoid treatment (2.186, 1.352–3.533), rash area (2.349, 1.083–5.095), HADS score (1.689, 1.112–2.564), GCH1 gene rs378641 genotype TT (2.136 1.314–3.473)	④	Low
Zhao (27)	Age≥50 years, coronary heart disease (1.651, 0.985–2.767), inciting factors for onset (3.680, 2.048–6.610), severe lesions (17.282, 7.677–38.905), NRS score (12.849, 5.393–30.611)	⑥	Low
Liao et al. (28)	Age (1.099, 1.004–1.204), rash area (1.528, 1.023–2.282)	④	Low
Tang et al. (29)	Diabetes duration ≥10 years (4.096, 1.759–10.082), GLUcv (5.234, 2.325–12.603), comorbidities (2.680, 1.143–6.567)	None	Low
Lin et al. (30)	Age, rash duration, NRS score, diabetes, history of malignant tumors, treatment duration, varicella-zoster virus lgM antibody level, serum neuron-specific enolase	③	Low
Wang et al. (15)	Age (4.43, 2.03–9.68), NRS score (28.14, 10.96–72.24), CCI score (1.87, 1.33–2.63), antiviral therapy (5.75, 1.13–29.21), immunosuppression (5.99, 2.03–17.63)	None	Unclear
Yang et al. (26)	Affected neural segments, age, VAS score, vesicle area, start time of nerve block therapy and pain nature	⑦	Unclear
Opstelten et al. (10)	Age (1.08, 1.04–1.12), VAS score (1.02, 1.01–1.03), rash severity (2.31, 1.16–4.58), rash duration (0.78, 0.64–0.97)	None	High
Cho et al. (11)	VAS score (1.583, 1.103–2.272), age (6.729, 1.193–37.946), S-LANSS score (1.156, 1.036–1.289)	②	High
Hashizume et al. (12)	Age, onset season, CCI score	③	High
Lu and Cheng (13)	Age (1.108, 1.057–1.162), VAS score (4.584, 2.247–9.351), underlying diseases (7.779, 2.461–24.591), treatment approaches (0.207, 0.065–0.666)	④	High
Li et al. (14)	Length of hospital stay, age, serum cholinesterase, MCHC, serum sodium, serum uric acid, TCO2, Bupleurum, WBC, TBA	None	High
Liu et al. (17)	Female (2.661, 1.136–6.230), age (3.026, 0.994–9.212), prodromal pain (2.711, 1.198–6.132), rash area (1.002, 1.001–1.003), VAS score (1.021, 1.002–1.041)	⑤	High
Lu et al. (18)	Age (1.909, 1.215–3.000), diabetes (2.294, 1.493–3.524), prodromal pain (1.193, 1.108–2.086), rash area (0.445, 0.337–1.075), VAS score (2.294, 1.493–3.524), initial treatment time (1.901, 1.023–3.532)	⑤	High
Zhang et al. (20)	Gender, age, VAS score, rash area, initial treatment time, anxiety, HZ site, HZ type, pain nature	None	High
Lu et al. (21)	N-acetyl-5-hydroxytryptamine, glucose, dehydroascorbic acid, isopropyl-β-thiogalactoside, 1,5-anhydro-d-sorbitol, glutamic acid	None	High
Wang et al. (24)	Age (3.522, 1.63–7.606), concomitant diabetes (2.182, 1.073–4.438), rash area (2.756, 1.426–5.327), prodromal pain (2.233, 1.216–4.099), NRS score (10.7224, 5.549–20.725)	⑤	High
Cai et al. (31)	Age (2.309, 1.163–4.660), NRS score (2.837, 1.294–6.275), platelet/lymphocyte ratio (1.015, 1.010–1.022)	⑤	High

VAS, Visual Analogue Scale; NRS, Numeric Rating Scale; NSE, neuron-specific enolase; TG, triglyceride; CRP, C-reactive protein; CCI, Charlson Comorbidity Index; S-LANSS, Self-completed Leeds Assessment of Neuropathic Symptoms and Signs pain scale; GLUcv, glucose coefficient of variation; GCH1, guanosine triphosphate cyclization hydrolase 1; MCHC, mean erythrocyte hemoglobin concentration; TCO_2_, total serum carbon dioxide; WBC, white blood cells; TBA, total bile acids; HADS, Hospital Anxiety and Depression Scale; ① scoring system; ② tool; ③ scoring table; ④ prediction model formula derived from regression coefficients of factors; ⑤ nomogram; ⑥ visualized using LIME tool; ⑦ SHAP plot.

### 3.6 Model performance

The area under the receiver operator characteristic curve (AUROC) for the models ranged from 0.714 to 0.980, with external validations conducted in 2 studies (15, 27). Wang et al. (15) applied a random forest model to predict 60 newly diagnosed patients with HZ, achieving an accuracy of 88.33% and a 95% confidence interval (CI) of 77.43–95.18%. The PHN risk prediction model constructed using the XGBoost algorithm by Zhao (27) demonstrated strong generalization and predictive performance in independent external validation datasets. External validation results showed that the model had an AUROC of 0.8377 (95% CI, 0.7660–0.9100) and an F1 score of 0.5143. Fourteen studies (10, 12, 16–19, 22–27, 30, 31) evaluated model calibration. The calibration curves indicated good agreement with actual outcomes, as supported by Hosmer–Lemeshow tests, which yielded *p*-values of >0.05. Eight studies (17–19, 24, 26, 27, 30, 31) assessed the clinical utility of the models.

## 4 Discussion

All PHN risk prediction models included in this study demonstrated AUROCs exceeding 0.7. Notably, 82.61% of the studies were conducted in China, suggesting the models’ favorable applicability to Chinese patients. However, 91.30% of the studies lacked external validation, highlighting the need for further investigation of their clinical utility. The high risk of bias in the included models was primarily due to homogeneous study populations, reliance on retrospective data, insufficient reporting of complex data handling and inadequate model validation.

The included studies reported PHN incidence rates of 17.30–48.00% domestically and 0.95–20.60% internationally. These disparities may be attributed to differences in population demographics, vaccination uptake, treatment levels, diagnostic standards and observation periods. Most studies collected data at or shortly after admission without accounting for factors such as treatment interventions or patients’ family and social contexts, resulting in considerable variability in predictive factors. Age, pain score and lesion area have been established as independent predictors of PHN, whereas the value of other factors remains unclear (32). For example, Xie et al. (33) meta-analysis found no association between gender and PHN onset, whereas Hao and Zhang (34) suggested that women are more likely to report severe pain and consequently are at higher risk of developing PHN. Patients with comorbidities such as diabetes or cancer, which compromise immune function, are susceptible to severe peripheral neural inflammation following HZ virus infection, leading to neural sensitization and subsequent PHN (35). However, few studies have conducted separate analyses of these comorbidities. Additionally, patients with PHN demonstrate neuroimaging changes (15), yet these factors have not been incorporated as potential predictors. With the growing adoption of genomic profiling techniques, there is potential for targeted therapies based on genotype variations (4), although acquiring such data may be challenging. Therefore, researchers are advised to systematically collect and collate previously reported predictive factors as candidate variables and, by integrating statistical methods with expert opinion, screen for clinically accessible factors to include in models for research purposes (5).

When compared with conventional modelling methods, machine learning shows clear superiority in handling factor selection and mitigating collinearity issues during the modelling process (5). Models constructed using different approaches demonstrate varying predictive performances, supporting the integration of multiple machine learning or deep learning techniques to improve prediction accuracy and identify the optimal model for predicting PHN. In the construction and validation of predictive models, considerations must extend beyond predictive accuracy and risk assessment effectiveness to include the models’ feasibility and practicality (5). Of the studies evaluated, 43.48% used data from model development to assess performance, only 8.70% underwent external validation and 65.22% did not evaluate clinical benefits. This disparity highlights that current PHN risk prediction models largely remain in the developmental stage, with insufficient assessment and validation for real-world clinical application. Therefore, further research is needed to validate and refine these models to ensure their accuracy and reliability in clinical settings.

Notwithstanding their inherent limitations, existing PHN risk prediction models remain essential tools for improving the management and prevention of PHN. Healthcare practitioners can use patient-specific characteristics to select appropriate predictive models, enabling the assessment and quantification of PHN risk in patients with HZ. Future research should prioritize prospective, multi-center studies with robust sample sizes. These studies should include age-subgroup analyses and employ machine learning methods to develop PHN prediction models tailored to the geriatric population. By integrating clinically accessible, objective and cost-effective factors, researchers can improve model performance evaluation and validation, presenting findings in a visually intuitive way. Furthermore, validating and updating existing models in line with diverse cultural contexts and clinical realities could achieve accurate predictive outcomes across different settings and populations.

In conclusion, this scoping review systematically elucidates the multifaceted characteristics of PHN risk prediction models. Although these models demonstrate promising predictive capabilities, they are characterized by a high risk of bias and remain in a developmental stage, necessitating further validation. Future research should prioritize enhancing the scientific rigor and standardization of study designs and model validation processes, aiming to develop tools with strong predictive performance and high clinical utility that provide reliable support for clinical practice. A limitation of this study is the predominance of domestically sourced models, with few international studies included. To address this gap, future researchers should expand database search scopes, conduct comparative analyses between domestic and international studies and foster more in-depth investigations.

## Data Availability

The original contributions presented in the study are included in the article/Supplementary material, further inquiries can be directed to the corresponding author.
