# Supplementary material for: A scoping review of models for predicting the risk of postherpetic neuralgia
Source: Front Med (Lausanne). 2025 Oct 3;12:1653680. doi: 10.3389/fmed.2025.1653680 (PMC12531067; doi:10.3389/fmed.2025.1653680)
Supplement: Supplementary file 1 [file Table_1.docx]

**Detailed search strategies for each database**

**1.China National Knowledge Infrastructure (CNKI) search strategy:**

（主题：带状疱疹 + 带状疱疹后神经痛 + 带状疱疹性神经痛）AND （主题：风险预测 + 预测模型 + 列线图 + 高危因素 + 影响因素）

**2.Wanfang**

 (主题:(带状疱疹 OR 带状疱疹后神经痛 OR 带状疱疹性神经痛) and 主题:(风险预测 OR 预测模型 OR 列线图 OR 高危因素 OR 影响因素))

**3.Vip**

题名或关键词=带状疱疹 OR 芾状疱疹后神经痛 OR 带状疱疹性神经痛 AND 題名或关键词=风险預测 OR 預測模型 OR 列线圏 OR 髙危因素 OR 影响因素

**4.Sinomed**

( "带状疱疹"[常用字段:智能] OR "带状疱疹后神经痛"[常用字段:智能] OR "带状疱疹性神经痛"[常用字段:智能]) AND( "风险预测"[常用字段:智能] OR "预测模型"[常用字段:智能] OR "列线图"[常用字段:智能] OR "高危因素"[常用字段:智能] OR "影响因素"[常用字段:智能])

**5.PubMed search strategy:**

#1 "Neuralgia, Postherpetic"[Mesh]) OR "Herpes Zoster"[Mesh]

#2 ((((risk prediction[Title/Abstract]) ) OR (predict*[Title/Abstract])) OR (model*[Title/Abstract])) OR (tool*[Title/Abstract])

#3 #1 AND #2

**6.Embase**

#1 'postherpetic neuralgia':ab,ti OR 'herpes zoster':ab,ti

#2 'risk prediction':ab,ti OR predict*:ab,ti OR model*:ab,ti OR tool*:ab,ti

#3 #1 AND #2

**7.Wos**

#1 postherpetic neuralgia (Topic) or herpes zoster (Topic)

#2 risk prediction (Topic) or predict* (Topic) or model* (Topic) or tool* (Topic)

#3 #1 AND #2

**8.Cochrane 97**

#1:(postherpetic neuralgia):ti,ab,kw OR (herpes zoster):ti,ab,kw

#2:(risk prediction):ti,ab,kw OR (predict*):ti,ab,kw OR (model*):ti,ab,kw AND (tool*):ti,ab,kw
